# Supplementary material for: N-N(+) Bond-Forming Intramolecular Cyclization of O-Tosyloxy β-Aminopropioamidoximes and Ion Exchange Reaction for the Synthesis of 2-Aminospiropyrazolilammonium Chlorides and Hexafluorophosphates
Source: Int J Mol Sci. 2023 Jul 11;24(14):11315. doi: 10.3390/ijms241411315 (PMC10379084; doi:10.3390/ijms241411315)
Supplement: Supplementary file 1 [file ijms-24-11315-s001.zip › ijms-2455464-supplementary.pdf]

Table S1. Crystal parameters and refinement details for **9**,**10**, **12**.

	**9**	**10**	**12**·H_2O
CCDC	2265181	2265182	2265183
Formula	$C_8H_{16}ClN_3$	$C_7H_{14}ClN_3O$	$C_{13}H_{22}Cl_2N_4O$
Fw	189.69	191.66	321.24
T, K	140	140	100
Crystal System	Monoclinic	Monoclinic	Monoclinic
Space group	$P2_1/n$	$P2_1/c$	C2/c
Z	4	4	8
a, Å	6.100(4)	12.752(4)	18.6123(5)
b, Å	11.059(7)	6.370(2)	6.8263(2)
c, Å	15.008(10)	11.611(4)	25.7555(7)
β, °	99.46(2)	98.890(9)	106.2140(10)
V, $Å^3$	998.7(11)	931.8(5)	3142.16(15)
D_{calc}, $g·cm^{-3}$	1.262	1.366	1.358
μ, mm^{-1}	0.336	0.369	0.415
F(000)	408	408	1360
No of measured refls.	8676	7388	21890
No of independent refls.	3102	2807	4831
No of observed refs. [I>2σ(I)]	1727	1724	4090
No of parameters	109	109	184
R1	0.0517	0.0562	0.0438
wR2	0.1369	0.1388	0.1001
GOF	1.017	0.973	1.017
Residual density, $e·Å^{-3}$ (d_{min}/d_{max})	0.290/-0.282	0.389/-0.303	0.510/-0.282

Table S2. Crystal parameters and refinement details for **13 – 16**.

	**13**	**14**	**15**	**16**
CCDC	2265184	2265185	2265186	2265187
Formula	$C_8H_{16}F_6N_3P$	$C_7H_{14}F_6N_3OP$	$C_7H_{14}F_6N_3PS$	$C_{13}H_{19}F_6N_4P$
Fw	299.21	301.18	317.24	376.29
T, K	140	140	295	100
Crystal System	Monoclinic	Orthorhombic	Triclinic	Monoclinic
Space group	C2/c	$P2_12_12_1$	P-1	$P2_1/n$
Z	8	4	2	8
a, Å	27.782(7)	8.649(3)	6.1161(12)	17.5027(3)
b, Å	6.1578(17)	10.594(4)	8.9032(18)	8.65640(10)
c, Å	14.965(4)	12.647(5)	11.827(3)	21.1288(4)
α, °	90	90	73.642(14)	90
β, °	110.172(7)	90	77.184(13)	100.9940(10)
γ, °	90	90	77.055(14)	90
V, $Å^3$	2403.2(11)	1158.7(7)	593.5(2)	3142.48(9)
D_{calc}, $g{\cdot}cm^{-3}$	1.654	1.726	1.775	1.591
μ, mm^{-1}	2.93	3.11	4.71	2.44
F(000)	1232	616	324	1552
No of measured refls.	11375	15501	8344	45941
No of independent refls.	3379	3617	3602	9659
No of observed refs. [I>2σ(I)]	1582	2258	976	8219
No of parameters	167	165	163	433
R1	0.0654	0.0738	0.1543	0.0525
wR2	0.1406	0.2000	0.2821	0.1501
GOF	0.948	0.961	0.927	1.013
Residual density, $e{\cdot}Å^{-3}$ (d_{min}/d_{max})	0.373/-0.347	0.586/-0.416	0.644/-0.640	1.137/-0.611
